# Supplementary material for: Magnetic resonance as a local probe for kagomé magnetism in Barlowite Cu4(OH)6FBr
Source: Sci Rep. 2018 Jul 18;8:10851. doi: 10.1038/s41598-018-29080-8 (PMC6052016; doi:10.1038/s41598-018-29080-8)
Supplement: Supplementary file 1 — Supplementary Information [file 41598_2018_29080_MOESM1_ESM.pdf]

Magnetic resonance as a local probe for kagomé magnetism in Barlowite $Cu_4(OH)_6FBr$

K. M. Ranjith[1,*], C. Klein[2], A. A. Tsirlin[3], H. Rosner[1], C. Krellner[2], and M. Baenitz[1]

[1] Max- Plank- Institute for Chemical Physics of Solids, 01187 Dresden, Germany
[2] Physikalisches Institut, Goethe-Universitat Frankfurt, 60438 Frankfurt, Germany
[3] Experimental Physics VI, Center for Electronic Correlations and Magnetism, Institute of Physics, University of Augsburg, 86135 Augsburg, Germany
[*] ranjith.kumar@cpfs.mpg.de

MAGNETIZATION

The temperature dependence of (1.8 K$\leq T \leq$ 300 K) magnetic susceptibility (χ) measured at different applied magnetic fields (H) is shown in Fig. S1(a). At high temperatures, $\chi(T)$ behaves in a Curie-Weiss manner and can be fitted by the expression

$$\chi = \chi_0 + \frac{C}{T - \theta_{\mathrm{CW}}}$$

where χ_0 is the temperature-independent contribution consists of diamagnetism of the core electron shells (χ_{core}) and Van-Vleck paramagnetism (χ_{VV}) of the open shells of the Cu^{2+} ions present in the sample. The second term is the Curie-Weiss (CW) law with the Curie-Weiss temperature (θ_{CW}) and curie constant $C = N_{\mathrm{A}}\mu_{\mathrm{eff}}^2/3k_{\mathrm{B}}$, where N_{A} is Avogadro's number, k_{B} is the Boltzmann constant, and μ_{eff} is the effective moment. The expression for effective moment is given by $\mu_{\mathrm{eff}} = g\sqrt{S(S+1)}\mu_{\mathrm{B}}$ where g is the Landé g-factor and S is the spin quantum number. Our fit to the 0.1 T data [Fig. S1(b)] in the high temperature regime yields $\chi_0 \simeq$ -1.19$\times10^{-6}$ $\mathrm{cm^3/mol}$, $C \simeq$0.45 $\mathrm{cm^3K/mol}$, and $\theta_{\mathrm{CW}} \simeq$ -145 K. The effective moment was calculated to be $\mu_{\mathrm{eff}} \simeq$ 1.9 μ_{B}/Cu. These values are in good agreement with the reported values.[1]

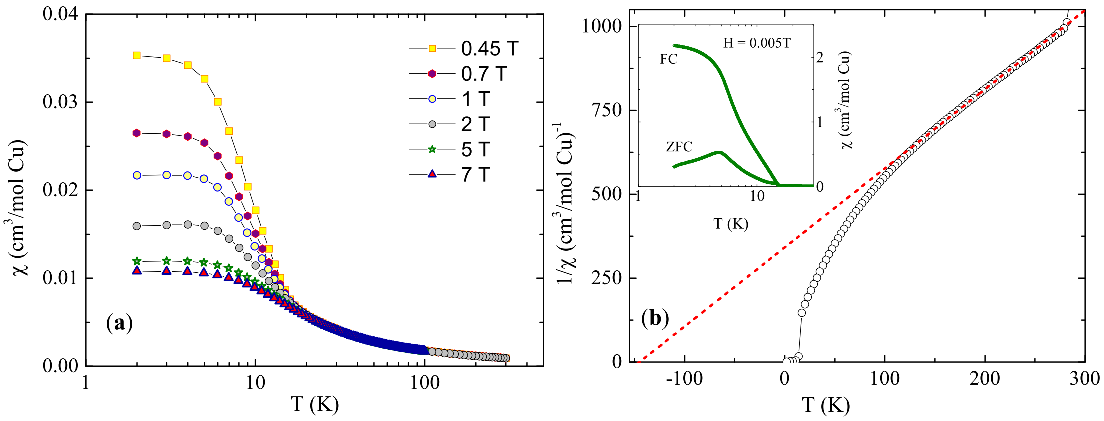

Fig. S1. (Color online) (a) The temperature dependence of magnetic susceptibility (χ) measured at different applied field. (b) Inverse magnetic susceptibility $1/\chi$ at 0.1 T and the solid line is the Curie-Weiss fit. The inset shows the field-cooled and zero-field-cooled susceptibility measured at 0.05 T

At low temperatures (1.8 K$\leq T \leq$ 30 K) zero-field-cooled (ZFC) and field-cooled (FC) magnetic susceptibility was measured at an applied field of 0.005 T. As Shown in the inset of Fig. S1(b), the ZFC-FC data shows a clear splitting at 15 K which indicates a phase transition to a long-range ordered state with a small ferromagnetic moment. Isothermal magnetization $M(H)$ measured at different temperatures are shown inf Fig. S2. It shows a hysteresis below T_{N} due to the interkagome Cu or Dzyaloshinskii-Moriya interaction (DMI).

REFERENCES

1. Han, T.-H., Singleton, J. & Schlueter, J. A. Barlowite: A spin-1/2 antiferromagnet with a geometrically perfect kagome motif. *Phys. Rev. Lett.* **113**, 227203 (2014).

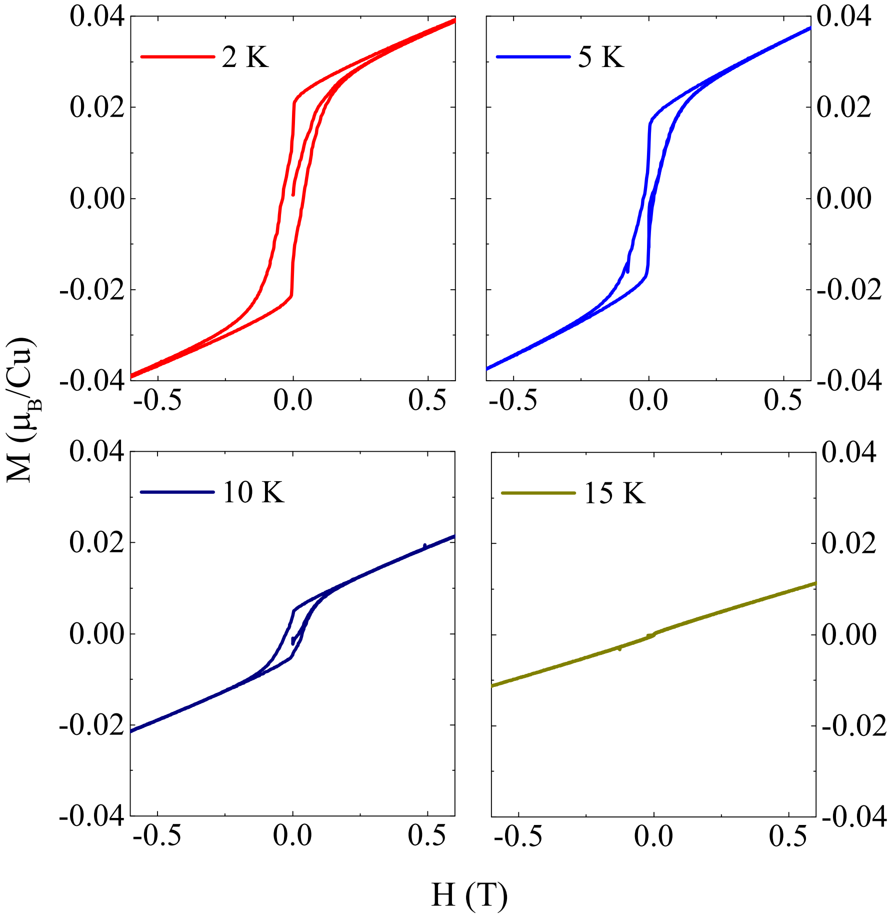

Fig. S2. (Color online) Magnetization isotherms (M vs. H) of measured at different temperatures.
